# Electronic Health Records and Antimicrobial Stewardship Research: a Narrative Review

**DOI:** 10.1007/s40471-021-00278-1

**Published:** 2022-07-21

**Authors:** Emma Rezel-Potts, Martin Gulliford

**Affiliations:** 1 School of Life Course & Population Sciences, King’s College London, Guy’s Campus, SE1 1UL London, UK; 2 NIHR Biomedical Research Centre at Guy’s and St Thomas’ Hospitals London, Great Maze Pond, London, SE1 9RT UK

**Keywords:** Antibiotic prescribing, Antimicrobial resistance, Antimicrobial stewardship, Electronic health records, Serious bacterial infections

## Abstract

**Purpose of Review:**

This review summarises epidemiological research using electronic health records (EHR) for antimicrobial stewardship.

**Recent Findings:**

EHRs enable surveillance of antibiotic utilisation and infection consultations. Prescribing for respiratory tract infections has declined in the UK following reduced consultation rates. Reductions in prescribing for skin and urinary tract infections have been less marked. Drug selection has improved and use of broad-spectrum antimicrobics reduced. Diagnoses of pneumonia, sepsis and bacterial endocarditis have increased in primary care. Analytical studies have quantified risks of serious bacterial infections following reduced antibiotic prescribing. EHRs are increasingly used in interventional studies including point-of-care trials and cluster randomised trials of quality improvement. Analytical and interventional studies indicate patient groups for whom antibiotic utilisation may be more safely reduced.

**Summary:**

EHRs offer opportunities for surveillance and interventions that engage practitioners in the effects of improved prescribing practices, with the potential for better outcomes with targeted study designs.

## Introduction

Antimicrobial resistance (AMR) has emerged as a global public health priority. Widespread excessive and inappropriate use of antimicrobials has contributed to the selection of strains of micro-organisms that carry mutations enabling them to survive exposure to antimicrobial drugs. Increasing resistance to currently available antimicrobials is compounded by the limited supply of new therapies in the drug development pipeline. If unchecked, the increase in AMR might threaten the foundations of modern medicine, ushering in a ‘post-antibiotic’ era in which infections are difficult or impossible to treat and routine medical procedures, surgeries and chemotherapy are associated with substantial risks. Globally, the number of AMR-associated deaths is predicted to rise from the current 700,000 per year to 10 million per year by 2050 [1]. The US Centers for Disease Control and Prevention (CDC) concluded that the post-antibiotic era is already here [2] with 2.8 million antibiotic-resistant infections and 35,000 deaths from resistant infections in the USA annually. In the European Union and European Economic Area in 2015, five types of antibiotic-resistant infections, including bloodstream infections, urinary tract infection, respiratory tract infections, surgical site infections and other infections, were estimated to account for 170 disability-adjusted life-years (DALYs) per 100,000 population [3].

There has been a global response to the threat of AMR including the World Health Organization’s *Global Action Plan on Antimicrobial Resistance* [4] and The European Commission’s *European Action Plan Against Antimicrobial Resistance in Europe* [5]. The 2016 independent review on AMR chaired by the economist Lord Jim O’Neill was instrumental in identifying actions required to reduce the demand for antibiotics and to increase their supply. The report has been credited with mobilising immediate and coordinated action across global institutions and governments [1]. In 2016, 193 Heads of State committed to implement the WHO’s Global Action plan at the United Nations (UN) General Assembly [6, 7].

AMR requires a multisectoral response aiming to reduce infections, improve methods for prevention, diagnosis and treatment of infection episodes, increase the supply of antimicrobials and reduce their consumption in agricultural and healthcare settings [1]. This review discusses antimicrobial stewardship in healthcare settings, particularly focusing on primary and ambulatory care where up to 80% of antibiotics are prescribed [8]. Antimicrobial stewardship has been defined as a set of ‘coordinated interventions designed to improve and measure the appropriate use of antimicrobial agents by promoting the selection of the optimal antimicrobial drug regimen including dosing, duration of therapy and route of administration’ [9] or, more simply as ‘[a] coherent set of actions which promote using antimicrobials responsibly’ [10].

The UK has some of the highest antibiotic prescribing rates in Europe and at least 20% of prescriptions are estimated to be inappropriate [11]. This is despite long-standing government-led advocacy of improved antimicrobial stewardship with successive action plans implemented in recent decades [12–14]. The UK’s 2013–2018 AMR strategy committed £615 million to AMR research, awareness and development activities [11, 13]. During this time (2014–2018), there was a 9% decrease in daily doses per 1000 inhabitants per day in general and dental practice [8]. However, this period also saw an increase in human antibiotic use in other community and hospital settings. In 2019, the UK government published both a new 5-year strategy [14] and a 20-year vision for AMR [11].

Electronic health record (EHR) systems have become important resources for surveillance, research and interventions contributing to AMR prevention. This review aims to summarise recent EHR-based AMR research studies, with a focus on primary care databases in the UK. This review provides an overview of studies which have used EHR systems to describe trends in antibiotic utilisation and monitor the effects of reduced prescribing on patient safety and for antibiotic prescribing feedback and audit interventions. We will also take a forward look at the development of EHR systems for more responsive tracking and management of infectious disease epidemics and for the advancement of effective antimicrobial stewardship interventions.

## Electronic Health Record Systems and Databases

The computerisation of patients’ clinical records has enabled the formation of large, population-wide databases that have become important resources for public health research. Primary care EHR systems are particularly relevant because family physicians and general practitioners (GPs) often act as gatekeepers to the healthcare system, providing longitudinal care over time and maintaining medical records that facilitate coordination of care received across community, primary and secondary care [15]. A systematic review identified 36 networks for primary care data collection incorporating the use of extraction tools, computing platforms and data linkage capabilities [16]. Major projects exist in Canada (the Canadian Primary Care Sentinel Surveillance Network, CPCSSN) [17], the USA (Veterans Health Administration) [18] and the Netherlands (Integrated Primary Care Information Project, IPCI, and Nivel-Primary Care Database, PCD) [19]. The UK primary care EHR databases, the Clinical Practice Research Datalink (CPRD) and The Health Improvement Network (THIN) are also well-known and contribute to many publications.

## The Health Improvement Network and Clinical Practice Research Datalink

The THIN database covers 6% of the UK population and is representative of population sociodemographic characteristics [20]. Estimates for consultation and prescribing rates based on THIN are comparable to national estimates [21]. The database includes anonymised data from approximately 12 million registered patients from almost 600 general practices. The CPRD has been operational for more than 30 years. Previously known as the General Practice Research Database (GPRD), it has generated upwards of 2500 research publications [22]. There are now two parallel CPRD databases depending on whether the contributing general practices use the Vision or Egton Medical Information Systems (EMIS) software [23]. General practices contributing to CPRD GOLD use Vision software [24]. The May 2020 release of CPRD included data for 18.8 million patients from 915 general practices, of whom 3.1 million, representing 4.7% of the UK population, were currently contributing. Only 21% of currently active CPRD GOLD general practices are in England with the remainder being in Scotland, Wales and Northern Ireland. The CPRD Aurum database now includes more than 1000 general practices, which are currently based in England only [25]. CPRD Aurum is now a larger database than CPRD GOLD but there is only limited research experience with CPRD Aurum to date. In an exploratory study, we found that analysis of CPRD Aurum gave similar estimates for antibiotic prescribing and infection consultation recording to CPRD GOLD [23]. Both CPRD databases offer data linkage to hospital episode and mortality registration data.

General practice systems in the UK store patient information, including symptoms, diagnoses and referrals, both as free text and Read codes but only coded data are generally available for research. Drug prescriptions, test results and lifestyle variables including smoking status, alcohol intake, height and weight measurements are also recorded [26]. The Read code system is a hierarchical coding system employed in the UK that provides codes for medical terminology relevant to primary care [27]. Over the last 2 years, the Read code classification has been replaced by the internationally recognised Snomed classification. The International Classification of Diseases 10^th^ revision (ICD-10) is not used in UK primary care, but the ICD-10 is used to code hospital episodes and mortality data. However, hospital episode data will transition to the Snomed classification over the next few years.

EHR databases offer several strengths for antimicrobial stewardship research. These are large, longitudinal databases of patient-level electronic health records that are subject to stringent data quality standards. They enable research which is speedier and less costly than studies requiring primary data collection [28]. Analysis of these databases can provide understanding of trends in antibiotic prescribing and bacterial infections that are nationally representative. Furthermore, both CPRD and THIN can be linked to other data sources at the patient level to enhance the completeness of recording of patient events across primary, secondary and community care and to improve data on disease covariates and health outcomes.

The limitations of EHR systems for AMR research include the potential for bias in the data as the recording of items largely depends upon clinical judgment, data items only being recorded when clinically indicated [29•]. Shallcross et al. point out that research using EHRs has the inherent limitation of relying on a dataset ‘devised for clinical management of patients rather than for the purpose of research’ [30]. Often with large datasets, even minimal effects are detectable and analyses can produce *P* values that are small, but prone to bias [29•]. Research using CPRD and THIN has found that prescriptions can be falsely mapped onto conditions leading to substantial differences in findings across papers which employ different methodologies and alternative ways of categorising Read codes. Up to a third of prescriptions in primary care may lack an informative diagnostic code [31••] and up to half of antibiotic prescriptions have no clear reason for prescription recorded [32••, 33•]. General practitioners regularly leave repeat antibiotic prescriptions uncoded and out-of-hours prescriptions from deputising services, walk-in centres and emergency care services are not generally recorded [32••]. Both CPRD and THIN have seen a decrease in participating practices which has reduced the sample sizes available for analysis of prescribing and consultations in recent years. Differences in outcomes between studies can also be an issue where there is variation in prescribing practices across participating GPs.

### Antibiotic Utilisation Studies

Early antibiotic utilisation studies using CPRD focused on acute respiratory tract infections (RTIs) as the group of conditions accounting for most antibiotic prescriptions. RTIs were commonly stated to account for 60% of antibiotic prescriptions in primary care, though recent studies cast doubt on this, as noted below. In the period 1994 to 2000, analysis of CPRD data from 108 general practices, with a population of 642,685 patients, showed there was a 35% reduction in the rate of RTI consultations from 422 per 100 registered patients per year to 273 per 1000 registered patient per year; this contributed to an overall 44% reduction in RTI antibiotic prescriptions [34]. This decline in RTI consultations has been confirmed in other national surveillance data in the UK [35], and is not fully explained. It is known that receiving an antibiotic prescription increases the chance that a patient will consult in future episodes of respiratory illness [36], so it is possible that there has been a process of de-medicalisation of common respiratory illness leading to lower consultation rates. However, the proportion of respiratory consultations at which antibiotics is prescribed has tended to remain fairly constant over time at just over 50% [37].

In response to the growing concern with AMR, a group at Public Health England explored the diagnoses associated with all antibiotic prescribing in primary care [33•]. Their study using the THIN database, using data for 2013 to 2015, found that 49% of antibiotic prescriptions were for respiratory infections, 20% for genitourinary tract infection, 16% for skin infections and 15% for other infections or infections at multiple sites. Nearly 20% of prescriptions were associated with uninformative codes (such as ‘telephone consultation’) or had no diagnostic codes recorded. Sun and Gulliford [32••] made similar observations using CPRD data from 2014 to 2017. Using a slightly different algorithm, their study found that RTIs accounted for 31% of antibiotic prescriptions, and genitourinary and skin infections accounted for 9% and 7% of antibiotic prescriptions, respectively. Some 15% of antibiotic prescriptions were not associated with diagnostic codes and the majority of these were repeat prescriptions. There were nearly 40% of antibiotic prescriptions with no informative diagnostic code recorded on the same day.

**Fig. 1:**
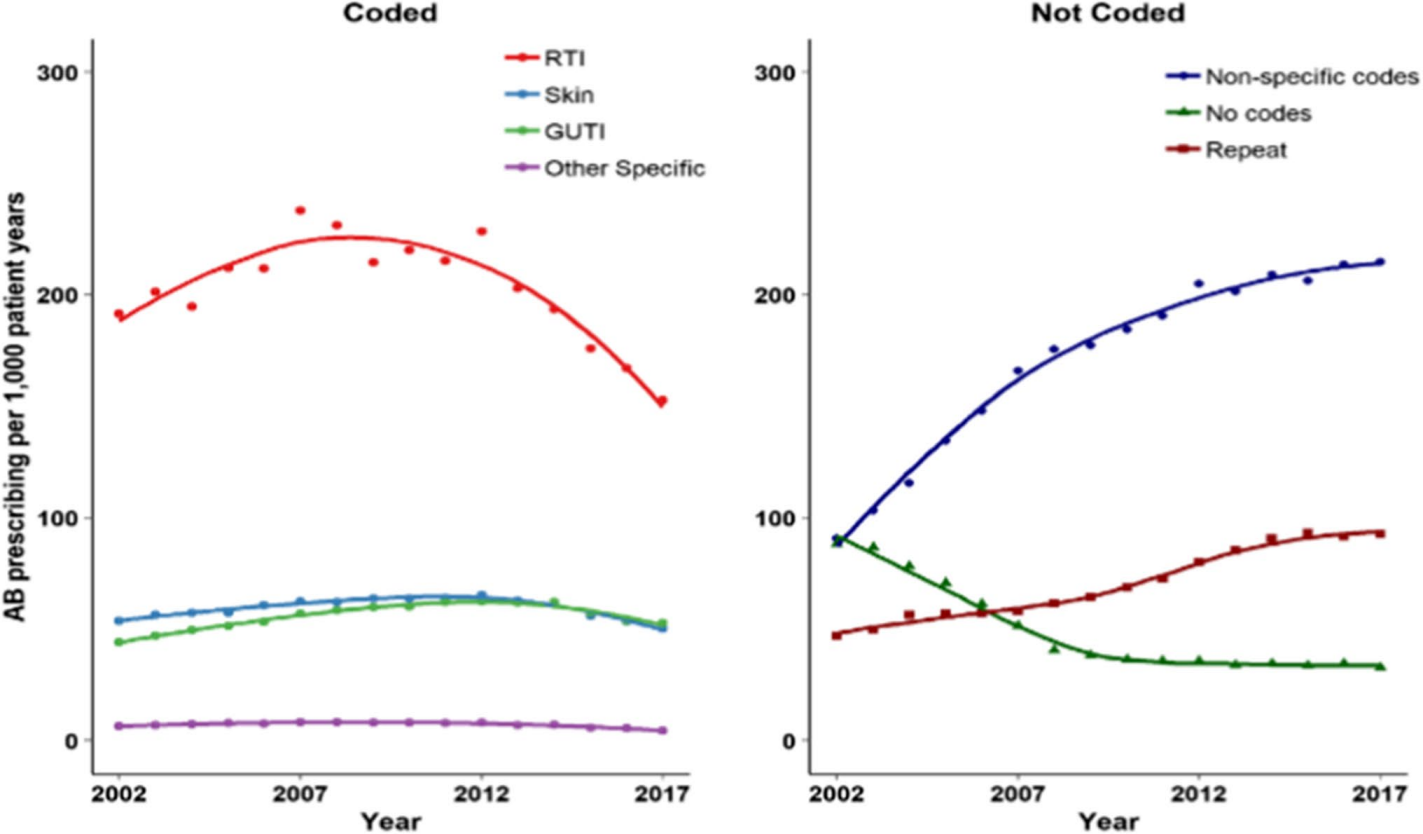
Trends in antibiotic prescribing for coded and uncoded indications (Gulliford et al. 2020). Gulliford MC, Sun X, Charlton J, Winter Jr, Bunce C, Boiko O et al. Serious Bacterial Infections and Antibiotic Prescribing in Primary Care: Cohort Study Using Electronic Health Records in the UK. BMJ Open. 2020;10(2): e036975 – CC BY

Figure 1 shows trends over time in antibiotic prescribing for these different indications [38••]. Antibiotic prescribing for respiratory infections has been declining since 2008, while antibiotic prescribing for genitourinary infections, skin infections and other specific infections has shown more modest declines since 2012. However, there have been compensatory increases in repeat antibiotic prescriptions and antibiotic prescriptions without clear indications recorded. These uncoded and poorly coded prescriptions may be markers of low quality of antibiotic prescribing; improving the recording of common infection episodes in primary care needs to be addressed as part of ongoing antimicrobial stewardship efforts [39].

These findings within primary care can be contextualised within broader, national trends. The English surveillance programme for antimicrobial resistance found that overall antibiotic consumption in the UK peaked in 2014 and subsequently declined to 2018 by 9% from 20.0 to 18.2 defined daily doses per 1000 population per day [8]. The decline was accounted for by reductions in prescribing in primary medical and dental care, while hospital antibiotic utilisation continued to increase. Dental practices in the UK account for about 10% of antibiotics prescribed in the community but these clinics do not have a unified electronic health record system and their antibiotic prescribing is difficult to monitor [40].

As well as a reduction in total antibiotic prescribing, antibiotic prescribing has become more selective with a decline in the use of broad-spectrum antibiotics and improved drug selection for treatment of urinary tract infections. In CPRD data, the annual relative reduction (RRR) for broad-spectrum β-lactam antibiotics was 9.3% (9.0–9.6%), which was higher than the RRR for all antibiotics which was 6.9% (95% confidence interval 6.6–7.1%) [32••]. This is indicative of changes in prescribing practices toward more targeted, narrow-spectrum antibiotics in line with guidance on prescribing for non-life-threatening conditions [41]. In England, nitrofurantoin is now recommended for urinary tract infection because of the growing level of resistance to trimethoprim, and this change is reflected in prescribing estimates from EHR studies.

Many of these descriptive studies have also investigated antibiotic use according to patient characteristics. Sun and Gulliford found that declines in antibiotic prescribing were similar for men and women and that the rate of decline was lower for those aged over 55 years compared to younger patients [32••]. Shallcross et al. found that half all the antibiotic prescription in THIN from 2011 to 2013 were for 9% of total patients [30]. The presence of any comorbidity increased the prescribing rate by 44% [adjusted incidence rate ratio (IRR) 1.44, 95% CI 1.43–1.45] and rate of prescribing to women was 62% higher than to men (adjusted IRR 1.62, 95% CI 1.62–1.63). These findings provide useful indications of where antimicrobial stewardship strategies could be better targeted and which patient groups may be at higher risk of serious bacterial infections.

### Analytical Epidemiological Studies and Safe Reduction of Antibiotic Use

Successful antimicrobial stewardship requires reducing inappropriate or unnecessary antibiotic use while ensuring continued effective management of suspected bacterial infections. This requires evaluation of the impact of antimicrobial stewardship policies on the occurrence of serious bacterial infections. In an early report, Petersen et al. [42] used EHRs to conduct a cohort study of patients presenting with RTIs. The study evaluated the association of antibiotic prescribing with the occurrence of mastoiditis after otitis media, peritonsillar abscess after sore throat and pneumonia after either upper respiratory tract infection or chest infection. Antibiotic prescribing at respiratory consultations was associated with lower risk of these complications but these were generally rare events and more than 4000 antibiotic prescriptions were required to prevent one complication. However, the risk of pneumonia was higher, especially following chest infection in people aged 65 years and over, for whom the number of antibiotic prescriptions needed to prevent one case of pneumonia was 39.

**Fig. 2:**
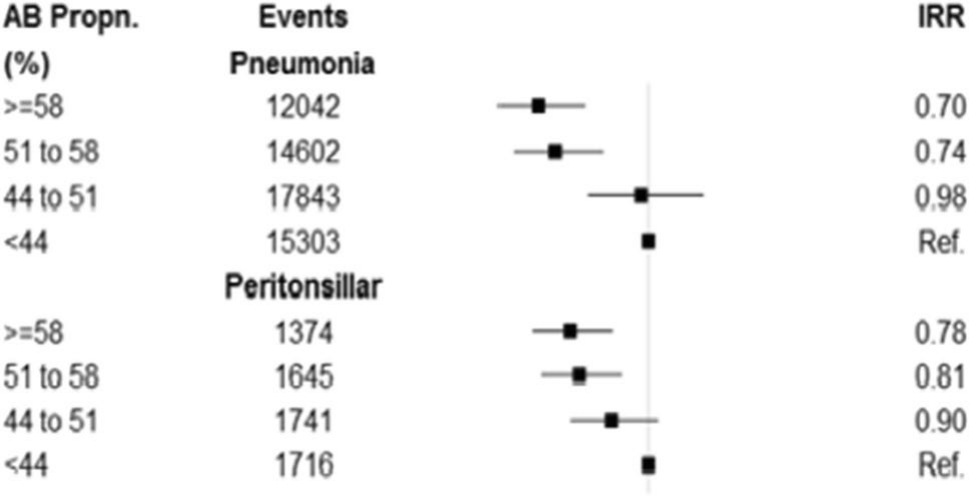
Association of pneumonia and peritonsillar abscess with proportion of RTI consultations with antibiotic prescribed (AB Propn) (Gulliford et al. 2016). Gulliford MC, Moore MV, Little P, Hay AD, Fox R, Prevost AT et al. Safety of reduced antibiotic prescribing for self limiting respiratory tract infections in primary care: cohort study using electronic health records. BMJ. 2016;354:i3410 – CC BY

A more recent analysis explored whether the incidence of pneumonia, peritonsillar abscess (PTA), mastoiditis, empyema, meningitis, intracranial abscess and Lemierre’s syndrome might be higher at general practices that prescribed fewer antibiotics for self-limiting RTIs [43]. The study found evidence that general practices with lower antibiotic prescribing for RTI had slightly higher risk of pneumonia and peritonsillar abscess (Fig. 2). A 10% reduction in antibiotic prescribing for RTI was estimated to be associated with a 12.8% (95% CI 7.8–17.5%) relative increase in pneumonia, and a 9.9% (5.6–14.0%) relative increase in PTA. The study estimated that in absolute terms if a general practice of average size reduces its antibiotic prescribing by 10%, this might result in one additional case of pneumonia per year and one additional case of PTA per decade. The study found no evidence that mastoiditis, empyema, meningitis, intracranial abscess and Lemierre’s syndrome might be more frequent at lower antibiotic prescribing practices. When the study was repeated with reference to total antibiotic prescribing, and not just prescribing for RTI, there was no evidence that lower total antibiotic prescribing was associated with increased risk of a range of serious bacterial infections [38••].

While evidence on patient safety and reduced prescribing has generally been reassuring with respect to RTIs, research on other indications has emphasised the need for caution among high-risk groups. Gharbi et al. used the CPRD (2007–2015) to analyse the association between antibiotics prescribed for UTIs and severe adverse outcomes, including sepsis, in elderly patients [44••]. After adjusting for covariates, the group of patients who were given a deferred antibiotic prescription and the group given no antibiotics at all were both significantly more likely to experience a bloodstream infection within 60 days follow-up compared with participants in the immediate antibiotics group (adjusted odd ratio 7.12, 95% CI 6.22–8.14 and 8.08, 7.12–9.16, respectively). The risk of all-cause mortality was significantly higher with deferred antibiotics and no antibiotics than with immediate antibiotics at any time during follow-up (adjusted hazard ratio 1.16, 95% CI 1.06–1.27 and 2.18, 2.04–2.33, respectively). Men older than 85 years were particularly at risk for both bloodstream infection and 60-day all-cause mortality.

### Use of EHRs in Interventional Studies

EHRs are increasingly being used to support interventional studies. EHRs enable trials to be conducted very efficiently, providing a sampling frame for general practice and participant recruitment and offering baseline and follow-up data for participant case-mix and outcome variables, as well as facilitating the delivery of electronic interventions. A key advantage of using EHRs in this way is that they enable trials to be conducted in usual care settings and including patients typical of those seen in routine clinical practice. These studies are sometimes referred to as ‘point-of-care’ trials and exemplify a very pragmatic attitude [45]. It is important that these studies give attention to the human–machine interface and ensure that interventions with behaviour change objectives, such as reduced antibiotic prescribing, are designed, pre-tested and implemented drawing on up-to-date behavioural science research methods [46].

EHR trials may be used to support antimicrobial stewardship programmes because they can be easily integrated into routine practice workflow and scaled up to provide population coverage. Most interventions have used clinical decision support tools, audit and feedback and educational interventions either alone or in combination. There is a growing evidence base to support the design of these interventions. A meta-regression analysis of RCTs using computerised clinical decision support tools found that interventions were more successful where physicians had to actively negate advice offered and where information was provided to both patients and clinicians [47]. Recent research has also contributed to understanding how and when performance feedback may be effective as a quality improvement strategy. Brown et al. [48] systematically reviewed 65 studies that evaluated 73 feedback interventions including both quantitative and qualitative literature. The review developed recommendations for effective practice and a Clinical Performance Feedback Intervention Theory (CP-FIT) [48]. An accompanying systematic review of 146 previous trials [49] provided guidance on how comparators can be selected and incorporated for performance feedback interventions.

A cluster RCT among 33 primary care practices within an integrated health care system in Pennsylvania, USA, found that a computer-assisted decision support and prescribing feedback intervention were effective in reducing prescribing for acute cough illness by nearly 15% [50]. Another cluster RCT, this time in Norwegian general practice, also led to improved antibiotic prescribing for RTIs in the intervention compared to the control arm (adjusted OR 0.72, 95% CI 0.61–0.84) [51]. These studies demonstrated the effectiveness of EHR-based interventions for antimicrobial stewardship, but also their potential to be resource intensive.

Our group conducted a point-of-care randomised trial to evaluate the effectiveness of an intervention using practice’s EHR systems to deliver decision support tools to reduce antibiotic prescribing for RTIs [52]. The decision support tools were installed remotely and delivered during the consultation, specifically activated when the family physician entered a medical code for the respiratory tract infection. It gave the clinician information for education and decision support, including an overview of antibiotic prescribing recommendations. The intervention effect was positive, but small, with poor utilisation of the intervention among some of the practices.

**Fig. 3:**
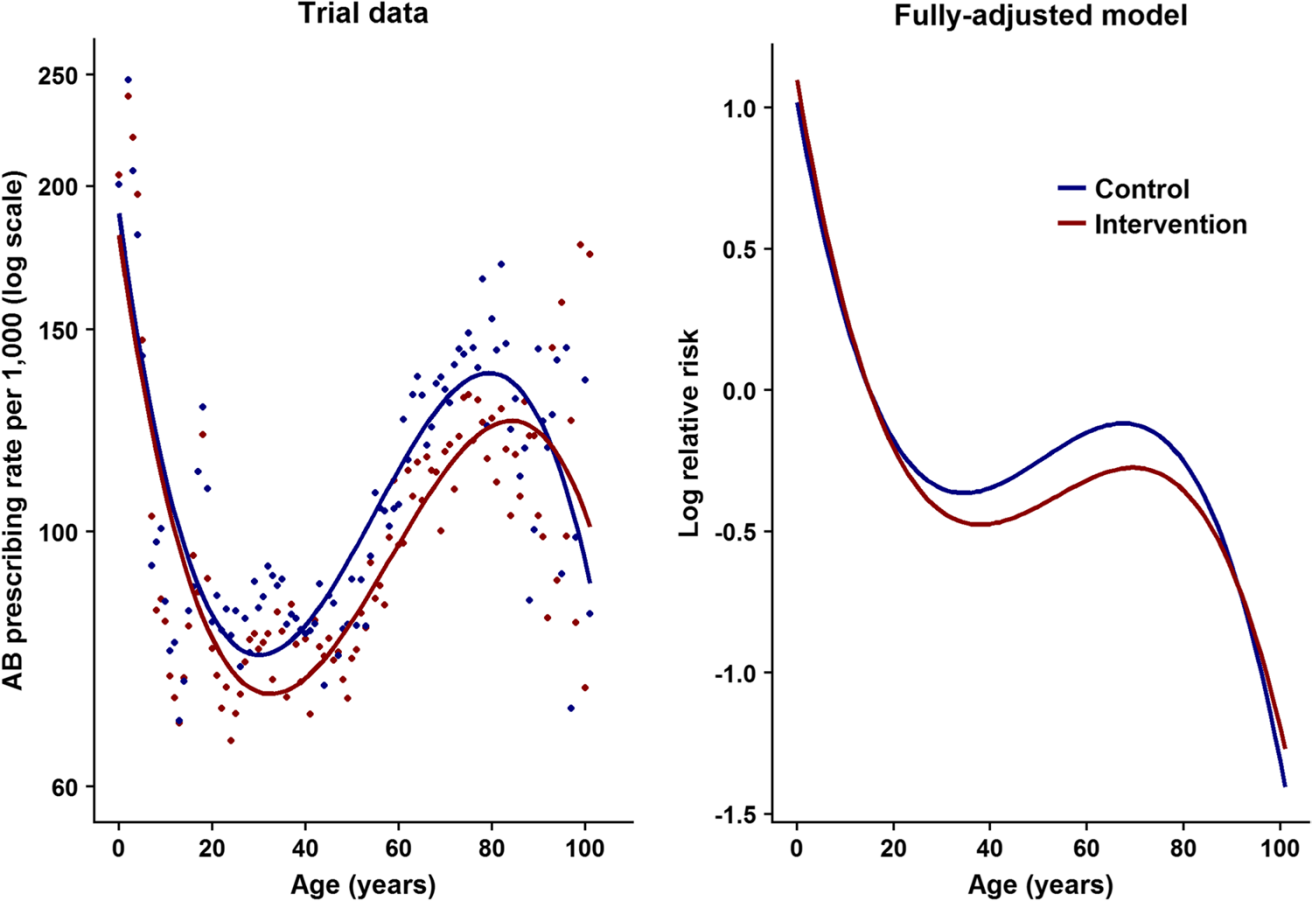
Age-related changes in antibiotic prescribing in the REDUCE trial (Gulliford et al. 2019). Gulliford M, Prevost A, Charlton J, Juszczyk D, Soames J, McDermott L et al. Effectiveness and safety of electronically-delivered prescribing feedback and decision support on antibiotic utilisation for respiratory illness in primary care. REDUCE cluster-randomised trial. BMJ. 2019;364:l236 – CC BY

In a subsequent study, we enhanced the intervention by feeding back information to practices on their own antibiotic prescribing [53••]. The 41 practices in the intervention arm received a short training webinar, automated monthly feedback reports of antibiotic prescribing and electronic decision support tools to inform appropriate prescribing during a 12-month period. Patients could access leaflets in print form or online. The adjusted rate ratio for antibiotic prescribing was 0.88 (95% CI 0.78–0.99, *P* = 0.04) and prescribing rates were 98.7 per 1000 patient years for intervention practices compared to 107.6 per 1000 patient years for practices in the control arm. There was no evidence of effect for those younger than 15 years or for those aged 85 years and over. This is exemplified in Fig. 3 which shows trial data by antibiotic prescribing rates for RTI by year of age with fitted polynomial curves suggesting lower antibiotic prescribing for RTI in the intervention arm for patients in their late teens to early eighties, but not among children or the very elderly.

### Forward Look

These cluster-RCTs have demonstrated that EHR systems can be actively engaged to enact antimicrobial stewardship strategies. Considering the limited impact on younger and older age groups, future intervention development would benefit from specific consideration of the needs of those who may be more vulnerable to serious bacterial infections. For example, purposely designed interventions have been reported as more effective for antibiotic prescribing to children [54]. EHR-based AMR interventions could also be extended to secondary care where there have not been sustained reductions in total antibiotic prescribing to date.

The COVID-19 global pandemic has brought the effects of unbridled infectious disease into sharp focus. After decades of chronic diseases dominating the public health agenda in high-income countries, this raises the question of whether prolonged prioritisation of infectious disease will follow such an unprecedented crisis. EHR systems have proved valuable in enabling healthcare systems to adapt to the rapidly evolving demands placed on them by the pandemic [55, 56]. EMIS software swiftly modified coding and introduced alert tracking and support of telemedicine among other interventions [57]. Future work should seek to develop EHR systems to be as responsive as possible for the tracking and management of infectious disease epidemics. The pandemic has highlighted the enormous public health value of the data held in large EHR systems. As recommended by Lord O’Neill in his 2016 report on infection prevention, control and surveillance, it is also important that private players in the EHR landscape are regulated and incentivised to enter the field of surveillance [58].

## Conclusion

Drug utilisation studies based on large datasets from EHR systems, including the CPRD and THIN, have provided representative estimates for national trends relevant to antimicrobial stewardship. Studies have shown declining antibiotic prescriptions, particularly for RTIs, corresponding with declines in consultation rates. Improved standardisation in coding, particularly for repeat prescriptions among patients with complex, long-term conditions, would enable more accurate monitoring. Studies using EHRs to investigate patient safety indicate that reduced prescribing is unlikely to increase rates of serious bacterial infections overall, but certain patient groups and indications continue to benefit from immediate prescriptions, such as elderly groups with UTIs. EHR systems are not only essential for monitoring of antibiotic prescriptions and rates of infection, but also offer unique opportunities to shape interventions that engage practitioners in the up-to-date, practice-level effects of improved prescribing practices, with the potential for better outcomes if intervention designs are more graduated and precise. The COVID-19 pandemic has brought global attention to the continued threat of infectious diseases. EHR systems should evolve for further AMR research and more responsive tracking for the management of infectious disease epidemics.
